# Surgery for Spine Disease and Intractable Pain

**DOI:** 10.3390/brainsci10020062

**Published:** 2020-01-24

**Authors:** Warren Boling

**Affiliations:** Department of Neurosurgery, Loma Linda University, Loma Linda, CA 92354, USA; WBoling@llu.edu

Painful conditions, particularly due to head pain, spinal disease, and neuropathic pain, are highly prevalent in modern society, resulting in a significant impact on the individual due to the disability of the condition and the direct cost of associated treatments. Additionally, indirect costs to society result from the loss of work productivity of the individual due to pain. For musculoskeletal pain alone, the related costs in the USA are estimated at $215 billion, of which 41% are from direct medical care, and the remaining are indirect costs associated with the condition [1]. The impact on society of head pain is manifest by the fact that this chief complaint is the fifth leading cause of visits to the Emergency Department and an even more common chief complaint for women aged 15–64 years [2].

In this Special Issue of Brain Sciences, the included manuscripts have explored several causes of pain and disability, their diagnosis, and treatment. Cantone et al. evaluated the disability that can result from mucopolysaccharidosis related cervical myelopathy [3]. The authors found that motor evoked potential measured from transcranial magnetic stimulation is a promising diagnostic tool to evaluate for early myelopathy before symptoms become overt. Trigeminal neuralgia is a severe and disabling face pain that can be effectively treated surgically in most people when medicines fail. Boling et al. reported their experience with different radiosurgery dosing protocols to target the root entry zone of the trigeminal nerve in the treatment of medically intractable trigeminal neuralgia [4]. The authors found that a higher dose regimen of 85Gy was the most effective and durable treatment for the facial pain. Spinal cord injury is an important cause of disability due to paralysis and loss of function as well as frequently associated neuropathic pain. Jermakowicz et al. evaluated the mechanisms underlying low-frequency electrical stimulation and the ability of stimulation to improve motor recovery and lessen allodynia related to spinal cord injury [5]. In the experiments performed, rats received a spinal cord injury at the cervical spinal level. The authors then identified that low-frequency electrical stimulation of the animals’ hindbrain nucleus raphe magnus resulted in decreased counts of cells related to neuro-inflammation and an increase in radial glia, which serve as a scaffold for neuronal migration in the area of injury. The findings of this study suggest a potential role for neuromodulatory therapies in the management of spinal cord injury and its sequelae. Hall et al. described an iatrogenic injury related to the treatment of a painful spine condition, then reviewed the literature of the treatment options available [6]. Vertebral artery injury is a rare but potentially devastating complication of surgery of the cervical spine. Surgery of the cervical spine is most commonly performed for pain related to instability, arthritic degenerative disease, or radicular pain resulting from nerve root compression. The authors reviewed treatment options and concluded that antiplatelet or anticoagulation were both options for the treatment of iatrogenic vertebral artery injury, and neither medical management approach has been found to be superior. Coccidioidomycosis is a fungal infectious disease caused by the Coccidioides species endemic to the Southwestern United States. Although this disease is rare, in endemic regions, Coccidioidomycosis can spread to the bone and spine, resulting in a destructive spinal disease. Ramanathan et al. described their institutions experience with spinal Coccidioidomycosis and reviewed the modern treatment approaches to this potentially devastating disease [7]. McGilvery et al. presented an unusual case of acute cervical disc herniation causing neurological deficit that resulted from self-manipulation of the cervical spine [8]. Heinrich et al. described a rare case of a primary osseous tumor of the spinal column of a 15 year old patient that required surgical decompression [9]. The authors described the surgical approach to a destructive lesion in C1 and the histopathology and radiology of a benign giant cell tumor of the spine. Chronic cluster headaches are a disabling form of headache characterized by severe unilateral pain in short-duration episodes, which are associated with ipsilateral autonomic symptoms primarily involving the temporal, supraorbital, and infraorbital head areas. Navarro-Fernández et al. described the value of a multimodal approach to treat this severe form of headache, which can include pharmacology, neurostimulation, and physiotherapy [10]. The authors presented a patient from their clinic who benefitted from a novel treatment combination of occipital nerve neurostimulation and physiotherapy approaches for chronic cluster headache. The authors went on to describe a mechanistic hypothesis for the headache resolution resulting from the different modes of treatment. The enormity of the topic of spine disease, pain, and disability could never be adequately covered in a single publication. However, this Special Issue provides valuable insights into a few of the most disabling pain conditions that affect mankind.
